# Supplementary material for: Mare Milk and Foal Plasma Fatty Acid Composition in Foals Born to Mares Fed Either Flax or Fish Oil During Late Gestation
Source: Animals (Basel). 2025 May 30;15(11):1612. doi: 10.3390/ani15111612 (PMC12153627; doi:10.3390/ani15111612)
Supplement: Supplementary file 1 [file animals-15-01612-s001.zip › animals-3618351-supplementary.pdf]

Table S1. Mean ± SEM morphometric measurements of unsupplemented control (CON) mares and mares supplemented daily with 242.4 g of fish oil (FO[1]) or 190 g of flax (FLAX[2]) beginning on day 310 of gestation.[3]

Day of Study[4]	Treatment			P-Value
	CON	FO	FLAX	
		Body Weight, kg		
GEST309	552 ± 14	520 ± 14	520 ± 18	0.23
PPD1	503 ± 10	500 ± 10	497 ± 13	0.94
PPD5	502 ± 9	493 ± 9	493 ± 12	0.77
		Body Fat, %		
GEST309	10.2 ± 0.4	10.5 ± 0.4	10.0 ± 0.6	0.75
PPD1	11.4 ± 0.4	11.4 ± 0.4	11.6 ± 0.5	0.85
PPD5	11.5 ± 0.3	11.4 ± 0.3	11.8 ± 0.4	0.81
		BCS, unit		
GEST309	6.7 ± 0.4	5.7 ± 0.4	5.7 ± 0.5	0.21
PPD1	6.1 ± 0.4	5.6 ± 0.4	5.3 ± 0.5	0.49
PPD5	5.9 ± 0.3	5.6 ± 0.3	5.5 ± 0.4	0.72

[1]DigestaWell Omega-3, Probiotech International, Saint-Hyacinthe, QC, Canada.

[2]Smart and Simple Flax, SmartPak, Plymouth, Massachusetts.

[3]Standard error of the mean

[4]Mares were evaluated prior to beginning treatment on day 310 of gestation (GEST309) and again on postpartum days 1 (PPD1) and 5 (PPD5).
